# The Need for Flexible Chemical Synthesis and How Dual-Function Materials Can Pave the Way

**DOI:** 10.1021/acscatal.3c00880

**Published:** 2023-05-15

**Authors:** Loukia-Pantzechroula Merkouri, Aysun Ipek Paksoy, Tomas Ramirez Reina, Melis S. Duyar

**Affiliations:** † School of Chemistry and Chemical Engineering, University of Surrey, Guildford GU2 7XH, United Kingdom; ‡ Inorganic Chemistry Department and Materials Sciences Institute, University of Seville-CSIC, 41092 Seville, Spain

**Keywords:** dual-function materials, integrated CO_2_ capture and catalytic utilization, switchable catalysis, flexible chemical synthesis, circular economy, direct air capture

## Abstract

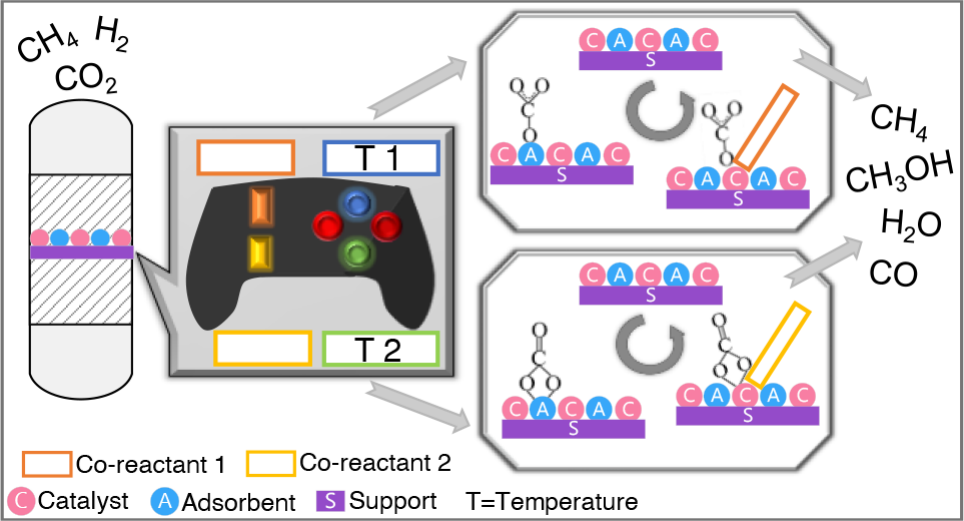

Since climate change keeps escalating, it is imperative that the increasing CO_2_ emissions be combated. Over recent years, research efforts have been aiming for the design and optimization of materials for CO_2_ capture and conversion to enable a circular economy. The uncertainties in the energy sector and the variations in supply and demand place an additional burden on the commercialization and implementation of these carbon capture and utilization technologies. Therefore, the scientific community needs to think out of the box if it is to find solutions to mitigate the effects of climate change. Flexible chemical synthesis can pave the way for tackling market uncertainties. The materials for flexible chemical synthesis function under a dynamic operation, and thus, they need to be studied as such. Dual-function materials are an emerging group of dynamic catalytic materials that integrate the CO_2_ capture and conversion steps. Hence, they can be used to allow some flexibility in the production of chemicals as a response to the changing energy sector. This Perspective highlights the necessity of flexible chemical synthesis by focusing on understanding the catalytic characteristics under a dynamic operation and by discussing the requirements for the optimization of materials at the nanoscale.

## 1 Introduction

Environmental and climate change due to greenhouse gas (GHG) emissions continues to pose a serious global threat. The alarming increase in anthropogenic CO_2_ emissions, i.e., 36.6 Gt in 2021 mainly due to fossil fuel consumption, despite the reduced industrial activities during Covid-19, calls for an urgent reduction in CO_2_.^1^ In fact, the United Nations have recently warned that the 1.5 °C ceiling imposed by the Paris Agreement cannot be achieved and, as things stand, the anticipated increase in temperature will be approximately 2.5 °C by the end of this century.^2^ The combination of using renewable energy sources with performing large scale carbon capture, utilization, and storage (CCUS) stands out as the only credible solution to mitigate the effects of carbon dioxide (CO_2_) emissions for the time being,^3^ with negative emission technologies, like direct air capture (DAC), also playing a role.^4−6^ To eliminate the carbon footprint of the chemical industry, it is essential to devise net carbon consuming processes through the catalytic conversion of carbon dioxide into carbon-containing chemicals and fuels, such as methanol, synthesis gas, synthetic natural gas, higher alcohols, and hydrocarbons, as a sustainable way of combating CO_2_ emissions while fostering a circular economy.^7^

Employing dual-function materials (DFMs) is a novel concept of integrated carbon capture and utilization (ICCU), as DFMs contain both adsorbent sites that can capture and concentrate CO_2_ on the surface and catalytic sites that convert the captured CO_2_ into a plethora of products. This integration of capture and utilization lowers the energy demands and thus the overall cost of the process due to process intensification. In addition to decoupling chemicals synthesis from fossil fuels, DFM technology provides an advantage on a process design level with its ability to catalytically convert CO_2_ from dilute streams without the added separation and transportation steps. DFMs can be implemented directly in any emission valorization scenario, including postcombustion and DAC applications. Especially in DAC applications, with commercialization plants now being the focus of several companies,^8^ DFMs allow the synthesis of chemicals to be carried out anywhere in the world (especially for on-site chemical production on demand), shifting toward a modular chemical industry with geographical freedom. This property makes it unique among the state-of-the-art CCUS technologies, such as the highly corrosive and energy-intensive amine systems.^9−12^ The technology can be applied to produce C1 products, such as methanol or methane locally, when renewable hydrogen (H_2_) becomes more widely available and, hence, reduce both economic and energy requirements. Methane (CH_4_), or synthetic natural gas, in particular, is very appealing due to the existing natural gas pipelines for transportation and the developments in catalyst design and process technology.^4^ On the other hand, methanol is another attractive chemical, given its versatility as fuel or as a building block for the production of alternative chemicals and fuels.^13^ Methanol spot price was USD 392/metric ton in Europe in June 2022,^14^ showing a clear trend of a growing market.^15^ Most importantly, it is feasible to produce methanol from renewable sources, as the George Olah plant has demonstrated.^16^ So far, DFMs have been evaluated in the production of methane and synthetic gas (CO and H_2_) by using H_2_, CH_4_, and ethane (C_2_H_6_) as coreactants,^9−11^ showcasing the wide range of CO_2_ utilization routes to which DFMs can be applicable. While advanced DFM pilot studies are still needed, the DFM concept based on the initial technology^17^ is being commercially developed, alongside a DAC demonstration plant being built in North Carolina, USA by Susteon.^18,19^

The decarbonization of chemical synthesis will rely on the use of renewable energy sources as well as the availability of green hydrogen as a sustainable reactant, especially in devising net zero or negative emission CO_2_ utilization processes. As we transition to a more sustainable future, it should be kept in mind that H_2_ and electricity generation fluctuate due to the variable nature of the renewable energy sources.^20^ Currently, the plants that produce renewable energy are connected to the national grid.^21^ The generation of renewable electricity affects the production of green hydrogen as renewable electricity is used for the production of green hydrogen via water electrolysis.^21,22^ The cost of electrolytic hydrogen is expected to decrease substantially in the long term, driven by the reductions that can be achieved from the declining costs of renewable electricity and the scaling up of electrolyzers and their manufacturing capacities.^23^ This anticipated price drop in green H_2_ will open up new horizons for the CCUS technologies, which heavily depend on it, by making them economically feasible.^22,24^ Currently, the price of green H_2_ is USD 3.0–7.5/kg^25^ and only 0.1% of the global H_2_ production is based on water electrolysis.^25^ This means that with the global H_2_ demand being at 90 Mt in 2020,^23^ only 0.09 Mt were produced via water electrolysis that year. Moreover, it is worth noting that valid concerns have been raised regarding the fresh H_2_O availability, especially due to microplastic pollution, and the environmental implication of water electrolysis.^26−28^ Perhaps alternatives based on biohydrogen production, i.e., from biomass and biowaste, should also play a significant role in meeting the needs for green hydrogen production on a large scale.^29,30^ The variability of the energy mix and the availability of green hydrogen as well as the costs of innovative green technologies pose risks to the development of CO_2_ utilization plants in the near future.^31−36^

Natural gas is generally seen as a low-carbon energy source that will help in the transition to the net zero future.^1^ The demand for natural gas fluctuates over the year since the need for heating purposes is higher during the winter. In the US, natural gas consumption reached ca. 3 billion cubic meters per day in the winter of 2019, but it decreased to ca. 2 billion cubic meters per day in the summer of the same year,^37^ signifying a 33% reduction in daily consumption. Likewise, in Europe, ca. 54 billion cubic meters of natural gas were consumed in January 2021, but only ca. 20 billion cubic meters were consumed in August 2021,^38^ meaning that there was a 63% reduction in monthly consumption. Apart from the seasonal change, there is also a change in natural gas consumption over the years. More specifically, ca. 2,000 billion cubic meters of natural gas were consumed in 1998 globally while ca. 4,000 billion cubic meters of natural gas were consumed in 2021.^39^ So, in just over 20 years, the global natural gas consumption doubled, denoting that there can be large fluctuations in natural gas supply and demand both over seasons and over years.

**Figure 1:**
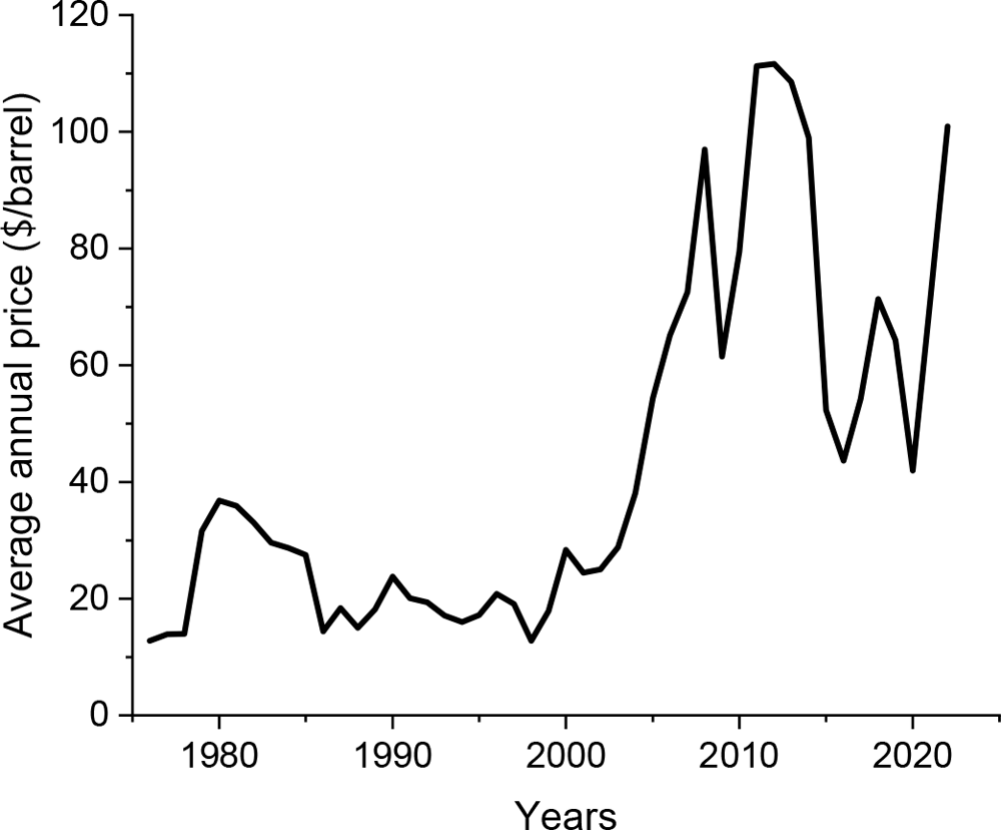
Change in Brent oil price throughout the years. Replotted with data given in ref (40).

In general, the energy sector depends on geopolitics. This is typically reflected in Brent Crude oil average prices shown in Figure 1.^40^ For instance, in 1980, Brent Crude oil price reached USD 37/barrel (up from ca. USD 14/barrel in the 1970s) due to the increase in its demand following the 1970s oil crisis. Similarly, its price skyrocketed in 2008, reaching USD 97/barrel because of the global financial crisis. The Covid-19 pandemic made the average Brent Crude oil prices plummet to USD 42/barrel in 2020 while the Europe Brent Spot price was USD 18/barrel in April 2020.^41^ In 2022, its price was USD 101/barrel,^40^ due to the cost-of-living and energy crises following the Covid-19 pandemic. Conclusively, the costs change over the years, depending on the supply and demand set by unexpected circumstances.

Given the continuing evolution of energy production and storage technologies, there are uncertainties that loom over the energy sector both in the short term and in the long term. Despite predictions of how the energy-related prices will behave, especially due to the development of green technologies, the energy sector is fraught with risks that put insupportable financial burden on the energy industries, turning new energy projects from profitable to unprofitable and vice versa.^42−44^ This market uncertainty, e.g., during Covid-19 pandemic,^44−46^ also affects the decision making for building new CCUS processes,^43,47,48^ and hence, future technologies and chemical plants should be prepared to adapt to current events so as to avoid an economic hardship or even an economic catastrophe.

One way of achieving this is to deliberately design for changes in supply and demand, creating an adaptable process. In this way, any given plant will be able to flexibly produce a plethora of chemicals and fuels while adapting to the supply and demand of the market. Currently, CCUS processes typically rely on catalytic materials to achieve selectivity and/or milder operating conditions. In a flexible chemical process that utilizes different feedstocks and produces a variety of chemicals, the catalysts themselves need to be “flexible” in terms of their operation. For such processes, a shift in paradigm is necessary; instead of searching for the most selective catalyst, it is possible to exploit versatility for flexible chemical synthesis. The ability of a material to catalyze multiple reactions by maintaining high activity and selectivity toward the individual reactions is a new approach within the CCUS technologies and one that is also being implemented in DFMs in an effort to reach a net-zero economy. Herein, we identify research needs in rational design of catalysts and DFMs to devise flexible processes for the transition to a carbon-negative economy.

## 2 Switchable Catalysis

### 2.1 Catalytic Characteristics and Needs

A switchable catalyst is defined as one that catalyzes different reactions when only the operating conditions are changed. Therefore, it can function effectively maintaining its high activity and selectivity upon switching from one reaction atmosphere to another, which is not a requirement for a conventional catalyst which is designed typically for one reaction. This provides flexibility in processes, and it is specifically important for the current unstable market for chemicals and fuels. Thus, a switchable catalyst can be exploited for supply and demand management.

In the CCUS context, switchable catalysts have been developed for the reduction of CO_2_ to a variety of products. Some of the most commonly investigated reactions of CO_2_ upgrading are CO_2_ methanation, reverse water–gas shift (RWGS), methanol (MeOH) synthesis, dry reforming of methane (DRM), bireforming of methane (BRM), and dry ethane reforming (DER). These reactions are presented below.^13,49−53^CO_2_ methanation:
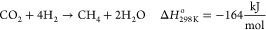
1RWGS:
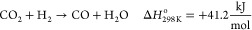
2MeOH synthesis:
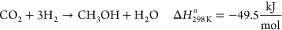
3DRM:
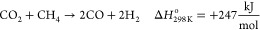
4BRM:
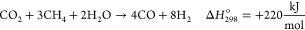
5DER:
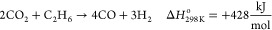
6

**Figure 2:**
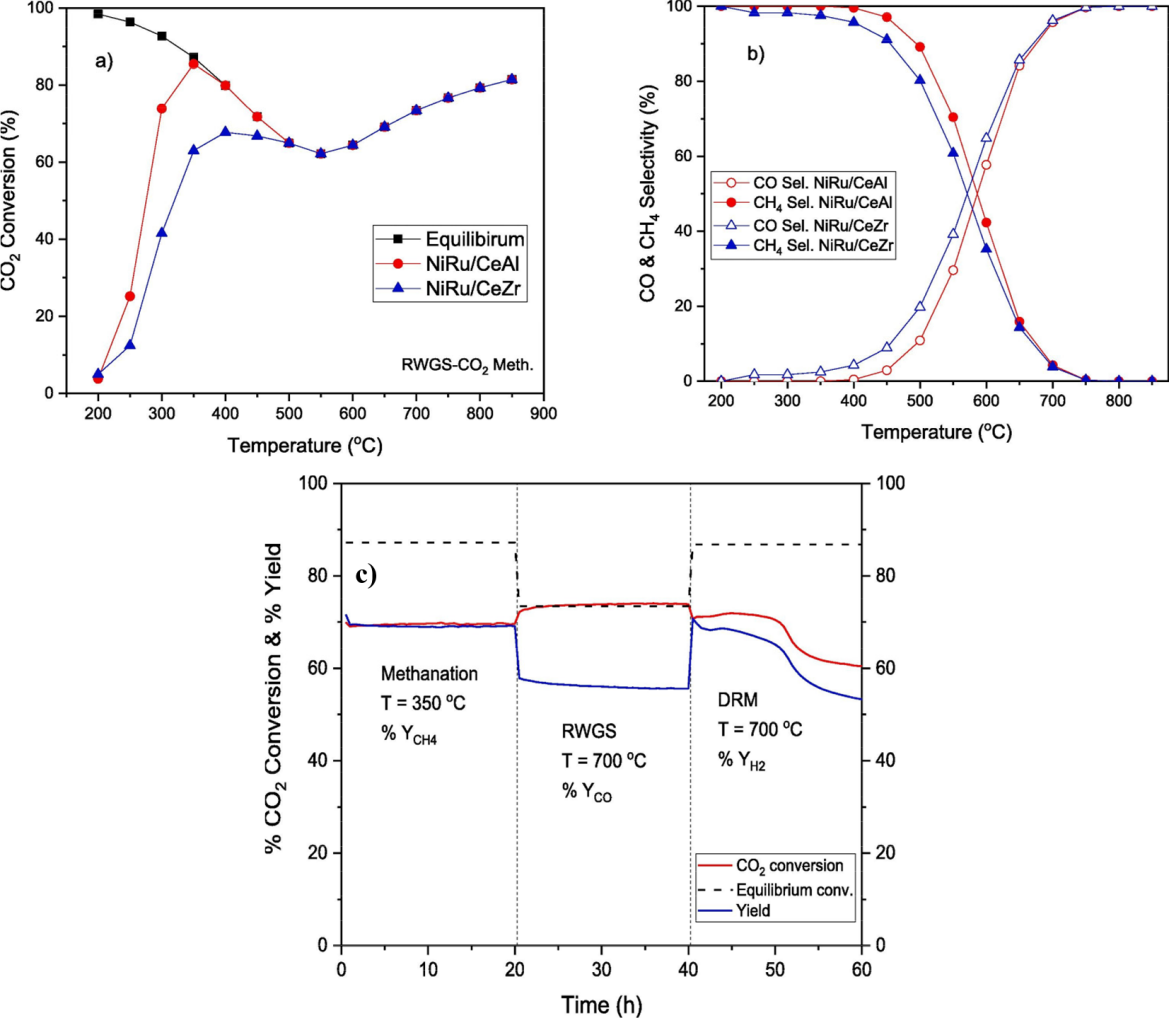
Versatile NiRu catalysts: (a) activity graph in CO_2_ methanation and RWGS, (b) selectivity graph in CO_2_ methanation and RWGS, and (c) stability graph in CO_2_ methanation, RWGS, and DRM. Reprinted with permission from ref (62). Copyright 2022 Elsevier.

**Figure 3:**
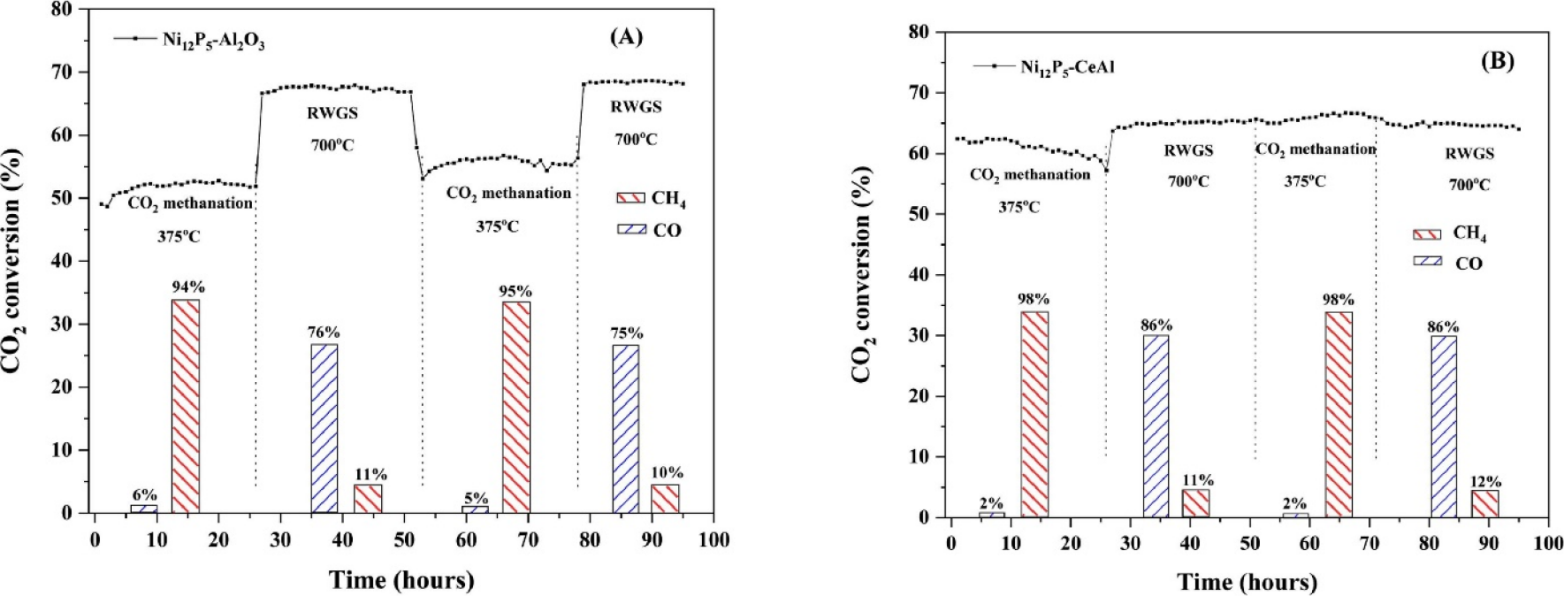
Stability test for versatile nickel phosphide catalysts in CO_2_ methanation and RWGS. Reprinted with permission from ref (63). Copyright 2022 Elsevier.

As can be seen, these reactions differ in terms of reaction enthalpy (endo/exothermicity) and coreactants, such as H_2_, H_2_O, CH_4_, and higher hydrocarbons. Consequently, it is feasible to alter reaction parameters to obtain different products. For instance, CO_2_ methanation and RWGS reactions have the same reactants (CO_2_ and H_2_), but the former is exothermic and the latter is endothermic. In recent years, a lot of attention has been given to designing selective CO_2_ hydrogenation catalysts by suppressing either CO or CH_4_ formation.^54−61^ While selective catalysts are important for several applications in the chemical industry, it is alternatively possible to design “switchable catalysts” that are highly active for reaching thermodynamic equilibrium. With a switchable catalyst for CO_2_ hydrogenation, it is possible to produce either CH_4_ or CO by adjusting the operating temperature. This variation in selectivity, depending on the reaction temperature, is depicted in Figure 2,^62^ since the same catalysts achieve 100% CH_4_ selectivity at low temperatures and 100% CO selectivity at high temperatures by maintaining high conversion values in both of them, i.e., 85% at 350 °C in CO_2_ methanation and 73% at 700 °C in RWGS.^62^ A similar trend can also be observed in Figure 3, showing the change in CO_2_ conversion and CO/CH_4_ selectivity upon temperature variation.^63^ Essentially, this is possible because both the CO_2_ methanation and the RWGS reactions are favored in different reaction conditions. For example, RWGS, DRM, BRM, and DER reactions are all endothermic reactions, and hence, high temperatures are usually required to obtain the desired product. However, they all have different reactant compositions. Consequently, switchable catalysts can also be designed to function efficiently in both reactions by simply changing the reactants’ mixture, as Figure 2c shows.^62^

One of the key aspects in switchable catalysis design is to select a component that is active in all desired reactions. For instance, when a switchable catalyst for the RWGS and DRM reactions is sought, it cannot solely consist of iron (Fe). Although iron (Fe) is excellent for the RWGS,^64^ it is inactive in the DRM^65^ and therefore it will not work as a switchable catalyst. Nickel (Ni), on the other hand, is one of the most promising DRM catalysts,^49,65^ while the addition of secondary metals to Ni-based catalysts is often employed to improve its catalytic stability in both DRM^53,66^ and RWGS.^67,68^ Thus, a combination of Ni and Fe would most probably be suitable when RWGS and DRM are the two targeted reactions.^56,69,70^ However, if the targeted reactions are the MeOH synthesis and the RWGS, suitable candidates are copper (Cu)-based ones.^71,72^ This is because Cu is very active in the RWGS,^51,64^ but it is also the commercial catalyst for the MeOH synthesis due to its ability to activate hydrogen.^71,73^ In this case, the catalyst would give us flexibility to produce CO or methanol by adjusting the pressure and feedstock H_2_/CO_2_ ratio. Essentially, the active component(s) of the switchable materials should be based on the desired reactions so as the activity and selectivity in the individual reactions are not compromised. In this respect, Ni/Al_2_O_3_, a typical CO_2_ methanation catalyst, is probably not a suitable candidate as a switchable catalyst for the CO_2_ methanation and RWGS reactions, because Ni/Al_2_O_3_ is known to promote the methanation reaction even at high temperatures, and 100% CO selectivity is not achieved unless extremely high temperatures are used.^64,74^ In general, the selection of the components of a switchable catalyst depends on the various reaction intermediates that are formed and on the reaction pathway each metal promotes. For example, Cu is one of the most suitable candidates for a switchable scenario involving the MeOH synthesis reaction due to the facilitation of the rate-determining hydrogenation of formate and dioxomethylene species.^73,75,76^ Typical active metals for DRM include Ni, Co, Ru, Rh, Pt, and Pd,^49,65,77^ for RWGS, Fe, Cu, Mo, Pt, Rh, Pd, and Ni,^51,64,73^ for CO_2_ methanation, Ru, Rh, Ni, Fe, Co, and Pd,^50,51,78^ and for MeOH synthesis, Cu, ZnO, Pd, Pt, Ga, and In_2_O_3_.^13,73,79^ Another aspect that needs to be taken into consideration is that switchable catalysis is a dynamic system; hence, it requires to be studied as such. The differences in catalytic needs for dynamic systems are illustrated in the studies of the power-to-gas technologies, in which the availability of renewable electricity fluctuates.^80,81^

The main purpose of switchable catalysis is to prevent a process from being financially unattractive due to variations in supply and demand. Therefore, although noble metals are known to work in all the aforementioned reactions,^49,64,82^ their cost hinders final commercialization (Ru = 16.7 €/g, Pt = 32.9 €/g, Pd = 53 €/g, and Rh = 415.8 €/g on 26 January 2023)^83^ and motivates the search for cheaper alternatives. It is worth mentioning that noble metal recycling is a well-established and viable technology, mitigating to a degree concerns around end of DFM life. In industrial catalytic processes that use noble metals, like fluid catalytic cracking (FCC) and selective catalytic reduction (SCR), high recycle efficiencies can be achieved, i.e., >99%.^84−87^ Hence, there is still room to incorporate noble metals to design highly effective DFM formulations but the technoeconomic and environmental impacts must be assessed according to the application. To date, most of the switchable materials tested are nickel-based (with some employing an alloying strategy to minimize the use of precious metals) due to nickel’s low cost, high activity, and stability in the desired reactions.^62,63,71,72,88^Table 1 summarizes all the switchable catalysts reported specifically with process flexibility targets in literature to date (although there are likely others that would show switchable behavior but have not been investigated specifically for this purpose).^62,63,88^

**Table 1:** Switchable Catalysts and Their Steady-State Catalytic Performance Reported in the Literature

	CO_2_ methanation		RWGS		DRM		MeOH synthesis		
catalyst	reaction conditions	CO_2_ conversion (%) at 350 °C	reaction conditions	CO_2_ conversion (%) at 700 °C	reaction conditions	CO_2_ conversion (%) at 700 °C	reaction conditions	CO_2_ conversion (%) at 150 °C	ref
Ni/CeZr	24 L·g^–1^·h^–1^	25	24 L·g^–1^·h^–1^	72	N/A	N/A	N/A	N/A	(88)
RuNi/CeZr	200–850 °C	53	200–850 °C	72					
FeNi/CeZr	CO_2_/H_2_ = 1/4	13	CO_2_/H_2_ = 1/4	71					
RuFeNi/CeZr	1.013 bar	41	1.013 bar	72					
NiRu/CeAl	24 L·g^–1^·h^–1^	85	24 L·g^–1^·h^–1^	75	24 L·g^–1^·h^–1^	85	N/A	N/A	(62)
NiRu/CeZr	200–850 °C	63	200–850 °C	73	550–850 °C	82			
	CO_2_/H_2_ = 1/4		CO_2_/H_2_ = 1/4		CO_2_/CH_4_ = 1/1				
	1.013 bar		1.013 bar		1.013 bar				
Ni_2_P/SiO_2_	12 L·g^–1^·h^–1^	5	12 L·g^–1^·h^–1^	60	N/A	N/A	N/A	N/A	(63)
Ni_12_P_5_/Al_2_O_3_	300–750 °C	28	300–750 °C	62					
Ni_12_P_5_/CeAl	52	CO_2_/H_2_ = 1/4	CO_2_/H_2_ = 1/4	70					
		1.013 bar	1.013 bar						
β-Mo_2_C Cu/Mo_2_C Cs/Mo_2_C	N/A	N/A	12 L·g^–1^·h^–1^	72	N/A	N/A	12 L·g^–1^·h^–1^	3.25	(71, 72)
Cu/Cs–Mo_2_C			400–750 °C	72			150 °C	5	
			CO_2_/H_2_ = 1/4	71			CO_2_/H_2_ = 1/3	3	
			20 bar	72			20 bar	4	

### 2.2 Design for Versatility vs Selectivity

In general, catalysts for CO_2_ conversion are designed with the main target of high selectivity toward one specific reaction. This is because the cost of product separation in the downstream operations accounts for 10–15% of the global energy consumption.^89^ Additionally, when hydrogenation reactions are considered, the presence of competing side reactions makes the process unattractive due to the consumption of highly valuable green hydrogen. Consequently, as a general rule higher selectivity prevents coreactants from being consumed by side reactions, lessens the need for downstream purification and product separation, and thus lowers the overall costs. The design of a highly selective catalyst is often associated with efforts to lower the cost and energy requirements. For instance, methanol synthesis is an exothermic process, typically requiring 50–100 bar and 200–300 °C.^90^ Research endeavors are targeted at lowering the energy and cost requirements by synthesizing an active and selective catalyst for low pressure operation.^73,79^ However, at low pressures, there are kinetic and thermodynamic limitations, and side reactions, like the RWGS, may take place.^13^ Therefore, in MeOH synthesis, selectivity plays an important role from an economic perspective because a less selective catalyst, operating at high pressures and requiring more downstream processing, will make the process economically unfeasible.

Product versatility is also very important since it can help to alter a common design parameter for the formation of C_1_ products and to use the same reactor for different purposes. In this context, let us assume that a CCU plant is placed downstream of a bioenergy plant that burns biomass for electricity production. During wintertime, when the demand for natural gas is high, the captured CO_2_ can react with H_2_ to produce synthetic natural gas, which can be sold for heating purposes. As time passes and summer comes, the need for natural gas drops, while the production of green hydrogen might increase depending on the local renewable energy production pattern. Therefore, the reactor temperature can be increased to promote the RWGS reaction. Thus, the syngas produced can be a valuable feedstock for the production of other chemicals. A downstream reactor may be used to produce methanol (CAMERE process)^91^ or higher hydrocarbons (Fischer–Tropsch synthesis)^92^ leading to final added-value products. Alternatively, methane production could be used for the seasonal storage of renewable energy, by producing methane during the summer months and using it during the winter months as a low emission fuel for residential heating. The exact schedule of operation can be adjusted to account for variability of renewable energy production and demand for various fuels over seasons and years. Evidently, there are many engineering challenges in the applicability of this “switchable chemical synthesis” approach, i.e., the transportation and storage of downstream products, safety regulations, and off-take agreements, resulting in re-engineering the way a chemical plant typically operates. For instance, handling CH_4_ and CO entails different challenges mainly because CH_4_ can be transported via pipelines, and CO would likely be used on site (but storage of high quantities would pose distinct safety concerns). On the other hand, the benefit of this approach will be that, once built, the CCU plant can respond to seasonal changes in product demand and green hydrogen availability and also allow facilities to produce chemicals on demand (such as CO) rather than having shipments which need to be stored in larger quantities. If the catalyst is designed for operation with various reactant choices (e.g., hydrogen and methane), CCU can further be derisked in the short term by operating with underutilized hydrocarbon feedstocks such as biogas.^31,33,34,46,93^ In this case, there is no need to change the catalyst, higher income is achieved since more valuable products can be produced in line with demand, and financial stability can be accomplished in the long term, perhaps by achieving more favorable terms when it comes to loans and investments.

## 3 Dual-Function Materials

Dual-function materials belong to a novel category of catalytic materials designed for integrated CO_2_ capture and utilization. Since their initial study,^17^ they have attracted a lot of attention from the scientific community.^9−12^ This is because they offer freedom of location (concentrated CO_2_ supply is not needed), application (postcombustion or direct air capture), and implementation as they can be placed in both a new and an existing plant with a relatively low footprint due to integration of separation and catalysis. DFMs do not require continuous operation or an existing carbon dioxide capture unit, and they eliminate the cost of CO_2_ compression and transportation (for offsite processing or storage), especially when the target product can be produced at atmospheric conditions.

Applying the concept of switchable catalysis in dual-function materials is motivated by the possibility of geographical freedom, wider application, implementation, energy usage, reactants availability, targeted products, and fewer supply and demand uncertainties. Switchable DFMs offer product versatility and financial shield. As DFMs and switchable DFMs are now past the proof of concept stage,^94^ it is important to devise a technological progress roadmap that relies on the rational design of catalytic materials for dynamic operation and process intensification at the nanoscale.

### 3.1 Materials Design for Dynamic Operation

Unlike most catalytic processes, DFMs will function under dynamic operation due to the integration of CO_2_ capture and conversion. This results in the requirement of different material design considerations from the static, or steady-state, operation.^9^ While designing active, selective, and stable catalysts is fundamental to any catalytic processes, dynamic operation presents unique challenges to achieve these goals, but it also offers opportunities for overcoming thermodynamic and kinetic limitations, as has been proposed in recent studies.^95−99^ All the reactions involve multiple sequential steps, including reactant adsorption, surface reaction, and product desorption, and each of them exhibits its own kinetics. By changing the reaction conditions, the energies of the surface-bound intermediates are fundamentally forced to shift to other more favorable energy conditions, reaching the optimum ones in each step separately. This energy asymmetry of the reaction profiles makes the dynamic catalytic reactions take place away from equilibrium,^95^ imposing fundamentally different constraints compared to steady-state reactions.

The surface of DFMs is continuously changing during capture and conversion due to the various forced reactor dynamic changes. Essentially, DFMs undergo continuous reduction and oxidation cycles during their lifetime, which impact their chemical structure, interaction with adsorbates, and long-term stability. Thus, it is important to design catalytic materials that do not deactivate due to the oxidizing conditions of CO_2_ capture. For instance, even though Ru is expensive, it is the most studied catalyst for CO_2_ capture and methanation owning to its ease of transformation between its reduced and oxidized states at low temperatures, ca. 150 °C. Even though oxidation is a known deactivation mechanism of Ru catalysts, the DFMs containing Ru are able to be reactivated upon exposure to hydrogen, therefore overcoming the oxidation issues in the O_2_-containing CO_2_ capture.^9,100^ Ni, on the other hand, is not as easily reduced and therefore, although it is a suitable, inexpensive, and earth abundant alternative to Ru for methanation under steady-state conditions, it presents a challenge for DFM applications.^101,102^ Alloying Ni and Ru has been shown to be a promising strategy for maintaining stability under cyclic redox conditions, while lowering the amount of expensive and rare element Ru in the formulation of DFMs.^94,101,103−105^ An alternative approach can be to incorporate catalysts that do not undergo deactivation under oxidizing conditions into DFMs. Metal oxide catalyzed reactions are therefore a promising area for exploration for DFM development. In addition, the effect of the realistic conditions should be addressed on a lab scale by observing the impact of typical impurities, such as O_2_, H_2_O, NO_*x*_, and SO_*x*_, on the DFM surface,^101,102,106−109^ and thus, their material design should be adapted and optimized.

**Figure 4:**
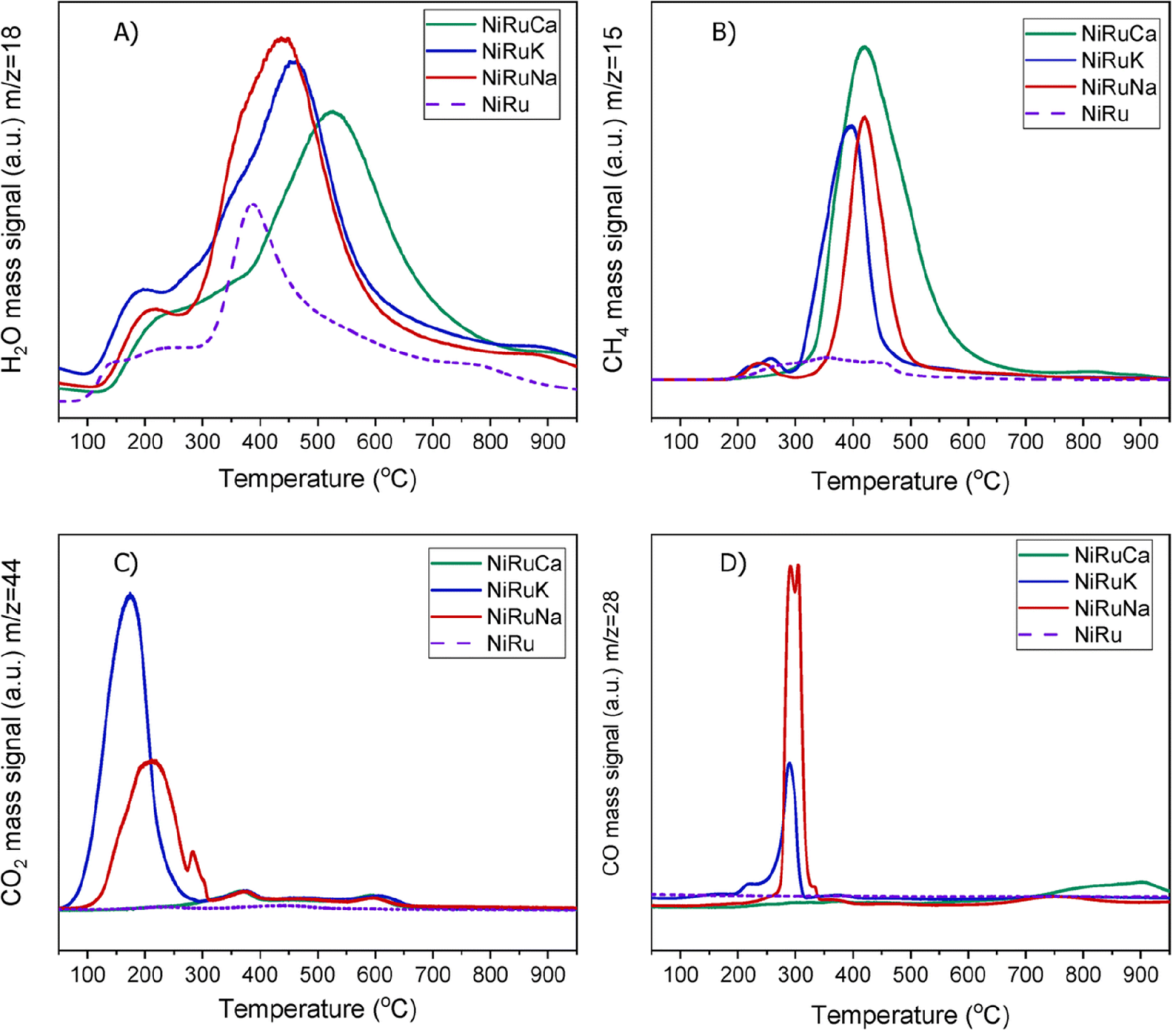
H_2_-TPR study for various NiRu DFMs, showing (A) H_2_O, (B) CH_4_, (C) CO_2_, and (D) CO signals. Reprinted with permission from ref (94). Copyright 2022 Royal Society of Chemistry.

Dynamic operation of DFMs can lead to a very different catalytic behavior due to the changing surface coverage of CO_2_, dynamic restructuring of the catalyst, and consequent changes in binding energies of the key reaction intermediates. Switchable DFMs demonstrate a very intriguing phenomenon whereby, following the capture of CO_2_ directly from the air, the hydrogenation of the surface species produces CO at lower temperatures and the selectivity switches to CH_4_ at higher temperatures,^94^ as Figure 4 illustrates. As can be observed, two DFMs consisting of sodium and potassium presented a CO peak at 300 °C and a CH_4_ peak at ca. 400 °C, but the third DFM consisting of calcium presented only a CH_4_ peak. Hence, there can be future opportunities in material design for the development of low-temperature RWGS applications. This finding is the exact opposite of what is observed under steady-state catalytic hydrogenation of CO_2_, where thermodynamics dictates that methanation is favored at lower temperatures, whereas reverse water–gas shift is favored at higher temperatures, as Figure 2b shows.

Moreover, surface–adsorbate interactions in DFMs are expected to change with time because they are influenced by the surface coverage of other molecules.^110,111^ Therefore, it is crucial to experimentally test the stability and cyclability of the DFMs so as to understand their behavior over time at different reaction atmospheres. An accurate prediction of the DFMs surface mechanism and active sites behavior in real chemistries via computational modeling is more complex than that in steady-state catalysis, but it is necessary to accelerate the scaling-up of the dynamic processes.^95,99^ Forming structure–activity relationships through *in situ* and *operando* characterization will provide key information in this regard, allowing one to tailor DFMs to applications. Regarding the experimental investigation of the DFM surface mechanism, there are only few diffuse reflectance infrared fourier transform spectroscopy (DRIFTS) studies that have been reported in the literature to date.^103,112−118^ Hence, it is important to make connections between structure and activity, which belong to an underexplored part of the DFM research. For example, if the reaction mechanism for low-temperature RWGS in Figure 4 is unravelled, research efforts can be focused on material design optimization.

In addition, the DFM synthesis technique is of particular significance because it allows us to control the dispersion of surface sites, which has a great effect on the CO_2_ adsorption capacity and conversion.^119^ If informed by mechanistic insight, a synthesis strategy can be developed to target specific improvements in performance, such as a higher CO_2_ capacity. However, the scalability of a synthesis technique is also important to consider (due to the massive need for CCU), especially in employing expensive synthesis processes compared to typical impregnation methods.^95^

Many other criteria for the DFM design, such as the pressure drop, require further attention.^4^ Consequently, the material design of the DFMs and the reactor setup need to be carefully adapted.^4^ Monoliths already play a key role in steady-state catalytic pollution control applications, like the NOx, and can be applied to DFMs in order to process large quantities of gas (especially for DAC applications) with minimal pressure drop. Microchannel reactors^120^ and fluidized bed reactors^121^ are also alternatives.

### 3.2 Process Intensification at the Nanoscale

**Figure 5:**
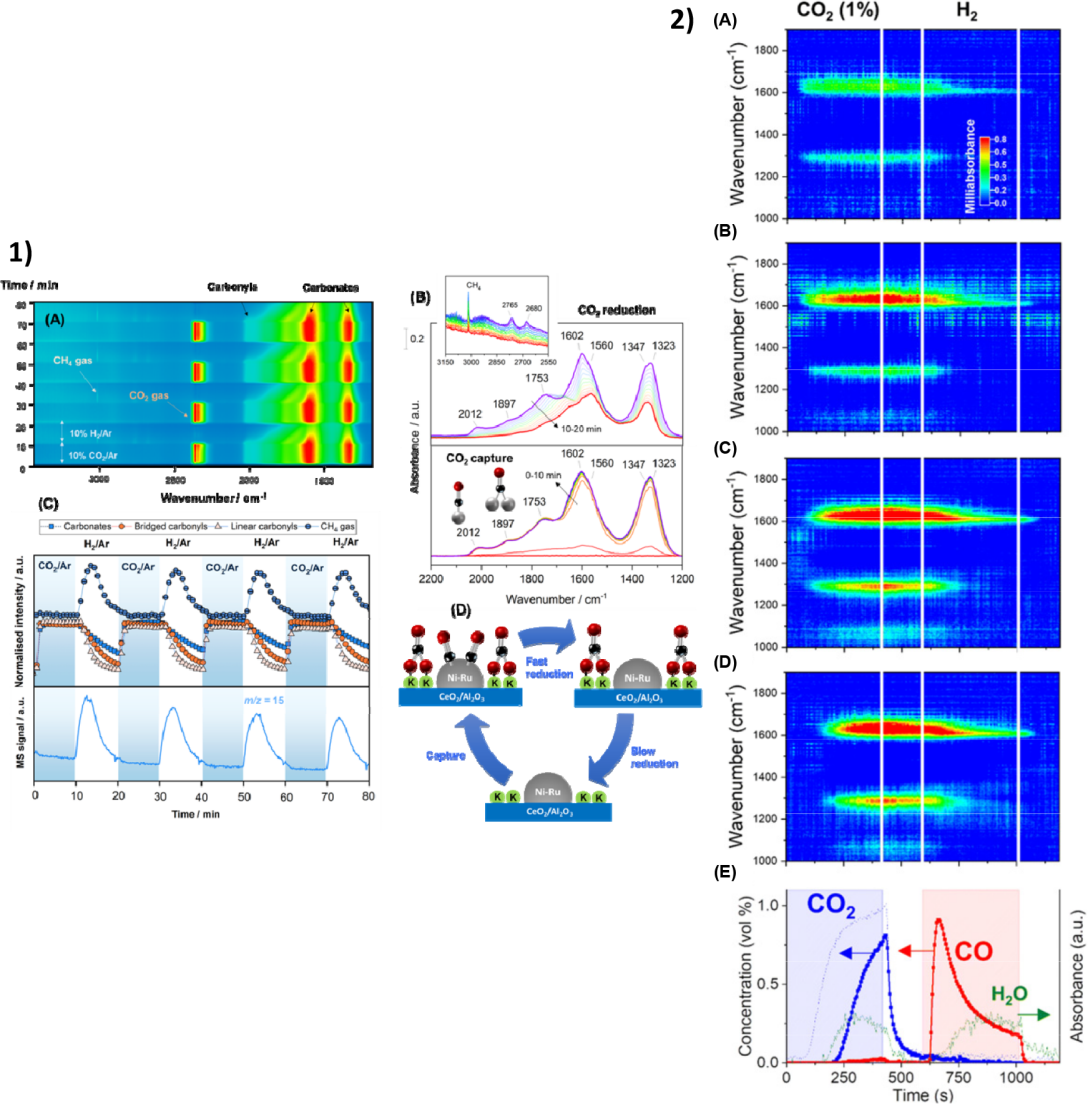
Mechanistic studies of DFMs for the integrated CO_2_ capture and conversion into (1) CH_4_ and (2) CO. (1A) Bidimensional representation of time-resolved DRIFTS spectra on a reduced NiRuK/CeAl sample at 250 °C during capture/reduction cycles, (1B) representative evolution of spectra during the first cycle of capture/reduction, (1C) evolution of selected IR bands and of a *m*/*z* = 15 (CH_4_) signal during the capture/reduction cycles, and (1D) illustrative sketch of the capture/reduction process on NiRuK/CeAl. (2) Dynamic evolution of surface species of Cu–K/γ-Al_2_O_3_ elucidated by *operando* DRIFTS during CCR at 350 °C in different positions of the catalyst bed from front (A) to back (D); (E) outlet gas composition at 350 °C. Reprinted with permission from refs (103 and 118). Copyright 2023 MDPI/Copyright 2022 Royal Society of Chemistry

A crucial parameter for the effective performance of the DFMs is the proximity of the adsorption and catalytic sites, which has been discussed from their onset.^17^ DFMs typically operate through a spillover mechanism whereby the CO_2_ for the catalytic reaction is supplied by a nearby adsorbent site, as the reactor is operated in a dynamic CO_2_ reduction mode.^17,103,122^ This spillover mechanism has already been observed in the literature.^103,112−114,118^Figure 5 shows the *operando* DRIFTS results of a nickel–ruthenium–potassium-based DFM in the CO_2_ methanation and of a copper–potassium-based DFM in the RWGS.^103,118^ In both cases, a fast reaction step occurs during the conversion step, followed by the spillover of the various carbonate species to the active metal sites for their subsequent conversion. Since the reaction pathway is unravelled by the *in situ* and *operando* characterization techniques, process intensification at the nanoscale can be achieved by developing materials with the most active species, further promoting a particular reaction route. Over the years, new experimental tools have emerged that assist in observing the structural changes as they are happening, e.g., X-ray absorption spectroscopy, ambient pressure X-ray photoelectron spectroscopy, identical location transmission electron microscopy and tomography, diffuse reflectance infrared Fourier transform spectroscopy, etc.^99^ The existing characterization tools as well as those in the pipeline are bound to play a major role not only in understanding DFMs but also in optimizing their design.

The close proximity of the adsorbent to the catalytic sites enhances the DFM performance, proving that it is more effective to have one material that can perform both CO_2_ capture and conversion instead of two separate ones. It has been reported that a physical mixture of a supported adsorbent and a supported active metal does not result in as large amounts of products as in coimpregnated adsorbent catalyst formulations.^17^ Nonetheless, if the sites are too close to one another, a negative effect might emerge, due to the active site coverage, which will hinder product formation.^119^ Consequently, the proximity and number of the adsorbent and catalytic sites are major factors affecting DFM performance, as many studies have already shown.^17,94,104,112,113,119,122−124^ The ideal DFM synthesis method should allow us to control how individual elements are placed, should result in optimum performance, should cause the least mass transfer issues, and should be low-cost. The optimization of both the adsorbent and the catalyst loading, due to potential surface coverage of the active catalytic sites, is also important.^17^

Another reason for optimizing the proximity of the sites is to ensure an efficient heat transfer. In general, DFMs make use of midtemperature adsorbents, which favor CO_2_ adsorption at intermediate temperatures. This is important because CO_2_ can be weakly adsorbed onto the surface, and thus, it can be easily regenerated.^9^ CO_2_ methanation, which is the most studied CO_2_ utilization reaction, and CO_2_ adsorption are both exothermic reactions. However, CO_2_ desorption is endothermic.^9,101^ Hence, coupling exothermic and endothermic reactions is energetically very desirable as it allows the DFMs to be operated isothermally, which is a big improvement over separate CO_2_ capture and utilization schemes, and the heat released from the CO_2_ methanation can be used to drive the endothermic CO_2_ desorption.^103^ However, in recent years, a large number of works have also demonstrated CO_2_ capture coupled with endothermic reactions, such as RWGS and DRM,^94,114,115,124−128^ where there are still benefits from a mass transfer point of view. In endothermic reaction applications of DFMs, the effects of heating or cooling during the cycles must be considered. It should also be noted that temperature gradients will exist, impacting the materials activity due to sintering. Sintering issues in DFMs have widely been reported in the literature for exothermic as well as endothermic reactions.^17,100,101,104,105,108,114,125,127,129−133^ The heat transfer issues therefore need to be resolved, particularly for scaling-up purposes. Perhaps the implementation of switchable DFMs on structured reactors, such as monolithic and microchannel reactors, could help to overcome heat transfer limitations.

In the design of DFMs, process intensification starts at the nanoscale. By better understanding the coverage and spillover mechanisms at play and the effects of impurities in the gas phase, as well as the adsorbent–catalyst interactions and their potentially promoting/deactivating effects on catalysis, it will be possible to design nanostructured DFMs offering superior performance than separate CO_2_ capture and catalysis units. Informed by *operando* characterization and theoretical understanding of structure–function relationships, we will be able to innovate in that regard, using synthesis strategies that enable the precise placement of different functionalities and control over the local catalytic environment, such as the nanoreactor design and single atom catalysis.^134−136^

## 4 Conclusion

This perspective emphasizes the importance of utilizing switchable dual function materials in today’s world of uncertainties. The variations in supply and demand, especially in the energy sector, place a large financial burden on the industry, turning otherwise economically feasible processes into unfeasible ones and vice versa. Flexible chemical synthesis combined with dual-function materials is an innovative way to protect future green processes. DFMs are an emerging group of catalytic materials that can introduce some flexibility in the chemicals production so as to respond to changing demands, unanticipated problems, and shifts in policy and decision-making. These materials fit into the broader vision of “on-demand” chemical production, using distributed sources of energy (like wind and solar) and locally available CO_2_-containing streams or atmospheric air. DFM design grounded on an understanding of the dynamic processes and process intensification at the nanoscale is crucial for achieving this vision of circular economy. It goes without saying that the accelerated growth of CO_2_ emissions calls for an urgent response from the scientific community, and this can only be achieved by perceiving the species interactions at a fundamental level and synthesizing the optimum materials for this dynamic operation. This will give rise to a self-sufficient market, unencumbered of financial insecurities, resulting in a new mindset in which CO_2_ from flue gases or directly from air will no longer be regarded as a threat but as a carbon pool for added value products manufacturing, complying with a circular economy strategy. If these challenges are met and overcome, flexible synthesis in dual-function materials will be a game changer for achieving net zero emissions.
